# Profiling the Succession of Bacterial Communities throughout the Life Stages of a Higher Termite *Nasutitermes arborum* (Termitidae, Nasutitermitinae) Using 16S rRNA Gene Pyrosequencing

**DOI:** 10.1371/journal.pone.0140014

**Published:** 2015-10-07

**Authors:** Michel Diouf, Virginie Roy, Philippe Mora, Sophie Frechault, Thomas Lefebvre, Vincent Hervé, Corinne Rouland-Lefèvre, Edouard Miambi

**Affiliations:** 1 Département SOLéO, Institut d’Ecologie et des Sciences de l’Environnement de Paris (IEES), Université Paris Est Créteil (U-PEC), Bâtiment P4, 61 avenue du Général de Gaulle, 94010, Créteil, France; 2 YNSECT Biotechnology Environment and Agro-Industry, Genopole-Campus 3, 4 rue Pierre Fontaine, 91058, Evry CEDEX, France; 3 INRA, Interactions Arbres – Microorganismes, UMR1136, F-54280, Champenoux, France; 4 Université de Lorraine, Interactions Arbres – Microorganismes, UMR1136, F-54500, Vandoeuvre-lès-Nancy, France; 5 UMR211 – Département SOLéO, IEES, Centre IRD France Nord, 32 avenue Henri Varagnat, 93143, Bondy, France; Universidade Federal do Rio de Janeiro, BRAZIL

## Abstract

Previous surveys of the gut microbiota of termites have been limited to the worker caste. Termite gut microbiota has been well documented over the last decades and consists mainly of lineages specific to the gut microbiome which are maintained across generations. Despite this intimate relationship, little is known of how symbionts are transmitted to each generation of the host, especially in higher termites where proctodeal feeding has never been reported. The bacterial succession across life stages of the wood-feeding higher termite *Nasutitermes arborum* was characterized by 16S rRNA gene deep sequencing. The microbial community in the eggs, mainly affiliated to Proteobacteria and Actinobacteria, was markedly different from the communities in the following developmental stages. In the first instar and last instar larvae and worker caste termites, Proteobacteria and Actinobacteria were less abundant than Firmicutes, Bacteroidetes, Spirochaetes, Fibrobacteres and the candidate phylum TG3 from the last instar larvae. Most of the representatives of these phyla (except Firmicutes) were identified as termite-gut specific lineages, although their relative abundances differed. The most salient difference between last instar larvae and worker caste termites was the very high proportion of Spirochaetes, most of which were affiliated to the *Treponema* Ic, Ia and If subclusters, in workers. The results suggest that termite symbionts are not transmitted from mother to offspring but become established by a gradual process allowing the offspring to have access to the bulk of the microbiota prior to the emergence of workers, and, therefore, presumably through social exchanges with nursing workers.

## Introduction

Gut microbial symbionts enable termites to play a key role in ecosystem processes such as carbon and nitrogen cycling. Termite-gut microbiota is very diverse and comprises many phylogenetic lineages that have been extensively documented in recent decades [1]. Previous studies have reported the presence of autochthonous lineages that are little affected by occasional changes of food or geographic location. Specific termite gut microbiota was found in the congeneric soil-feeding species *C*. *ortognathus* from a Kenyan grassland and *C*. *niokolensis* from a Senegalese savannah [2]. The presence of gut-specific Actinobacteria has been reported in the wood-feeding termite species, *Nasutitermes corniger* Motschulsky, collected from various geographical sites [3]. As termite gut symbionts have coevolved with termites, their community structure is basically consistent within a genus [4]. In higher termites, the gut of wood-feeding species that feed on lignocellulose components is mainly colonized by Spirochaetes and members of the Fibrobacteres phylum and related candidate phylum TG3 while the gut community of soil-feeders which thrive on nitrogen-rich components is dominated by Firmicutes. In fungus-growing species, the dominant phyla are Bacteroidetes and Firmicutes. Both host phylogeny and diet can be important determinants of the bacterial community structure in termite guts [5–7]

The maintenance of symbioses through generations of the hosts depends on reliable symbiont transmission. It has been shown that the maintenance of host-specific flagellates in lower termites was achieved via vertical transmission during trophallaxis [8]. Little is known about the transmission routes of digestive symbionts in higher termites and most hypotheses are based on transmission processes in lower termites. Recently, a molecular survey of the gut microbiomes of specimens representing higher and lower termite genera showed that vertical inheritance was the primary force shaping termite gut microbiota [9]. As in most previous studies on termite gut microbiota, this molecular survey focused only on the worker caste. No studies have so far been published on the gut communities of larval instars or on the successive changes in the gut microbiota during the development of higher termites. By monitoring the eggs, first and second instars (L1 and L2), and adult workers of a wood-feeding termite species in a comparative analysis, the present study specifically addresses the question of whether the gut microbiota of the last instars originates from maternal transmission through the egg or early inoculation of the offspring. Eggs were included in the present study to give a comprehensive insight into the successive changes of the microbiota during termite development and determine whether the gut microbiota of later instars originates from maternal transmission through the eggs or early inoculation of the offspring. Pyrosequencing of the 16S rRNA gene was used to characterize the variation in the taxonomic composition of the bacterial communities associated with the life stages. To assess the role played by the transmission of symbionts to offspring in the codiversification of the gut microbiota in a higher wood-feeding termite host, 16S rRNA gene based clone libraries generating longer sequences were used to determine the phylogenetic relationships between the sequences of the most dominant lineages in the final development stages and those retrieved from the gut of allopatric *Nasutitermes* species. The present study set out to characterize changes and assess the differences in microbial communities throughout the various development stages of a wood-feeding termite species *Nasutitermes arborum* (Smeathman) to gain a better understanding of the bacterial colonization of the gut by their symbionts.

## Materials and Methods

### Termite sampling

A higher wood-feeding termite species, *N*. *arborum* (Smeathman) was used in this study. The genus *Nasutitermes* Dudley, 1890 is a key taxon widespread in all tropical areas, with 243 species currently described. *Nasutitermes* species are wood-feeding termites and their post-embryonic development has been well established. A nest of *N*. *arborum* (Termitidae, Nasutitermitinae) was collected in the Republic of the Congo (Brazzaville), in the Mayombe forest within UNESCO-Man and the Biosphere Reserve, in the district of Mvouti (4°14’S, 12°26’E), with the permission of Dieudonné MBOUMBA, the Administrative Head of the district of Mvouti (Congo). This field study did not involve endangered or protected species. The nest was kept in a termitarium at the Institut de Recherche pour le Développement (IRD) in Pointe Noire (Congo). Termites were identified on the basis of morphological criteria and in comparison with laboratory collections by Dr Alain Robert, entomologist and termite expert. The nest was partially broken to collect the eggs, larvae and adult workers. The two larval instars were distinguished using the standard morphometric criteria for the *Nasutitermes* genus [10,11].

### DNA extraction

Triplicate samples of 50 eggs, 30 individuals of each larval stage and 25 workers were used. The eggs were surface-sterilized by dipping them twice in 70% ethanol for 1 min, followed by five rinses in sterile water. The whole gut of termite larvae and workers was removed aseptically using fine sterile forceps under a bacteriological hood and pooled for each replicate in 1.5 ml sterile microtubes.

Guts and eggs were first crushed using a polypropylene micro pestle in 1.5 ml microtubes. The DNA was then extracted using the DNeasy Blood & Tissue Kit (QIAGEN, QIAGEN GmbH, D.40724 Hilden, Germany) in accordance with manufacturer’s instructions. The final DNA concentration was determined by electrophoresis in a 1% agarose gel, with a quantitative DNA ladder (High DNA Mass Ladder, Invitrogen, 5791 Van Allen Way Carlbad, CA 92008) for comparison. Prior to pyrosequencing analyses, the DNA concentration of the samples was quantified photometrically using a ND–1000 Spectrophotometer (NanoDrop products, Wilmington, USA).

### Pyrosequencing and processing of 16S rRNA gene sequence

The purified DNA from the triplicates was pooled for pyrosequencing. Aliquots with similar DNA concentrations were sent to the Research and Testing Laboratory (Lubbock, TX, USA) for sequencing. A DNA fragment spanning the V1-V3 variable regions of the 16S rRNA genes was amplified using the eubacterial primers 28F and 519R and pyrosequenced using a Roche 454 FLX pyrosequencer. The resulting sequences were analyzed using Mothur v.1.33.3 [12]. Pyrosequencing reads were processed using the method described by Schloss [13]. Sequencing error was reduced using an implementation of the AmpliconNoise algorithm and low-quality sequences were removed (minimum length 200 bp, allowing 1 mismatch to the barcode, 2 mismatches to the primer, and homopolymers no longer than 8 bp). Sequences were then trimmed to keep only high quality reads (Q ≥ 35). Chimeras were removed using the chimera.uchime mothur command. Singletons were included in the analysis. Sequences were aligned and classified according to the SILVA bacterial SSU reference database v.102 [14]. They were then assigned to genus-level phylotypes using the naive Bayes classifier implemented in Mothur and clustered into operational taxonomic units (OTU) using the average neighbor algorithm and a sequence identity cutoff of 97%. The Greengenes database [15], and the DictDb reference database v.2.3 [6] were used for the taxonomic assignment of OTUs. The shared OTUs (97% sequence identity) between life stages (eggs, L2 larvae and workers) were determined using the Venn diagram package implemented in Mothur. The Venny 2.0.2 software was used to plot resulting data. Relative abundance of each group of OTUs was added manually. Sequences are available in NCBI Sequence Read Archive under BioSample accession ID SAMN03114856 and SAMN03114878 to SAMN03114880 associated with BioProjects PRJNA270400- PRJNA270403. In each pyrotag library, the relative abundance of an OTU is the percentage of reads included in this OTU with respect to the total number of reads. The relative abundance of each taxon in a given sample is the sum of abundances of all the OTUs included.

### Clone libraries and phylogenetic analysis

The near-full-length 16S rRNA genes were amplified using the 27F/1390R primers [16]. PCR reactions were performed in 25 μL reaction mixtures containing 15.2 μl Taq polymerase Master Mix (Qiagen), 0.25 μM of each primer and 50 ng of template DNA as described by Thongaram et al [16] using a Prime Elite thermal cycler (TECHNE®, Bibby Scientific Limited, Staffordshire, UK). Amplicons were gel-purified using the GFX^TM^ Purification Kit (GE Healthcare, Buckinghamshire, UK) and cloned into the pCR4-TOPO vector using the TOPO TA cloning kit (Invitrogen), following the manufacturer’s instructions. White clones were screened for the presence of the expected insert by PCR amplification using the vector-specific primers M13F-20/M13-26R. For each sample, 70 positive clones were double-sequenced at Beckman Coulter Genomics (Takeley, CM22 6TA Essex United Kingdom). The sequences were quality checked and processed in the same way as for pyrotag sequences. A phylogenetic analysis was performed using OTUs classified within the most representative phylum (Spirochaetes). A nucleotide Blast search was run and the five closest sequences imported for each OTU. Other Spirochaetes 16S rRNA gene sequences were randomly imported. The redundant sequences were removed and the sequences were aligned using SILVA SINA (www.arb-silva.de/aligner/) with the reference dataset SILVA SSU Ref NR [17]. The alignment was improved manually by removing conserved gaps and ambiguously aligned regions. The phylogenetic tree was reconstructed using the Maximum Likelihood method implemented in MEGA v 6.0 [18] and the Tamura-Nei model with 1000 bootstraps.

## Results

### Analysis of pyrosequencing data and taxonomic classification

454 pyrosequencing produced a total of 33351 raw pyrotag reads for the four samples. After quality filtering and removal of chimera, 18310 high quality reads were retrieved. These ranged from 1470 to 5990 sequences per sample (Table 1) clustered at 97% sequence identity into 78 bacterial OTUs. The OTUs affiliated to *Wolbachia* spp. covered 77.2% of reads from eggs, 98.6% in L1 larvae, 53% in L2 Larvae and less than 1% in workers. As *Wolbachia* spp. are endosymbionts and not digestive symbionts per se in Isoptera, all representative *Wolbachia* OTUs were subsequently excluded from analysis. Consequently, as only 50 non-Wolbachia reads remained from L1 larvae, this development stage was not included for downstream analysis. For the remaining stages (eggs, L2 larvae and workers), in order to avoid bias attached to an unbalanced library size, the data were normalized based on the egg size library that had the lower number of sequences (1367). The same number of sequences was, therefore, randomly subsampled in L2 larvae and workers. The resulting sequences from these three stages were assigned to 687 OTUs unequally distributed depending on the stage. The DictDB database [6] provided the best taxonomic assignment of OTUs and classified them into 18 bacterial phyla. Most of the OTUs fell within the following phyla: Proteobacteria (31.46%), Spirochaetes (27.94%), Bacteroidetes (15.44%), Firmicutes (7.27%), Actinobacteria (6.02%), Fibrobacteres (4.71%), Candidate phylum TG3 (4.54%), Acidobacteria (0.98%) and Synergistetes (0.29%). The remaining OTUs fell within 9 minor phyla, each accounting for less than 1% of the reads with fewer than 5 OTUs per stage: Cyanobacteria, Caldiserica, Candidate division SR1, Chlorobi, Chloroflexi, Deferribacteres, Elusimicrobia, Planctomycetes, and Verrucomicrobia. The number of OTUs in the eggs was more than 5 times lower than in the gut of L2 larvae and workers. Furthermore, the workers and L2 larvae had far more genus and phylum level taxa, suggesting an overall higher bacterial diversity in the last developmental stages than in eggs.

**Table 1:** Sampling depth and number of taxa at OTU level (at 97% identity), at genus level and phylum level by 16S rRNA gene pyrosequencing from the various life stages of *N*. *arborum*. For eggs, L2 larvae and workers, the numbers of taxa are based on a sub-sample of 1367 reads.

	Eggs	L1 larvae	L2 Larvae	Workers	Total
**Sampling depth**					
Quality filtered reads	5990	3678	7172	1470	18310
Non-Wolbachia reads	1367	50	3371	1458	6246
**Taxonomic composition**					
Number of phylum-level taxa	7	7*	13	17	18**
Number of genus-level taxa	58	19*	93	97	178
Number of unique OTUs (97% identity)	72	24*	297	357	687**

*For L1 larvae, they are based on 50 sequences excluding *Wolbachia*. They are given for illustration only and are not included in the total for the taxonomic composition, which refers to unique taxa.

** For the taxonomic composition, the total number of taxa at the phylum, genus and OTU levels is the sum of the individual taxa from the three pyrotag libraries, taking account of the taxa common to different stages.

### Changes in the bacterial community structure throughout life stages of *N*. *arborum*

**Fig 1:**
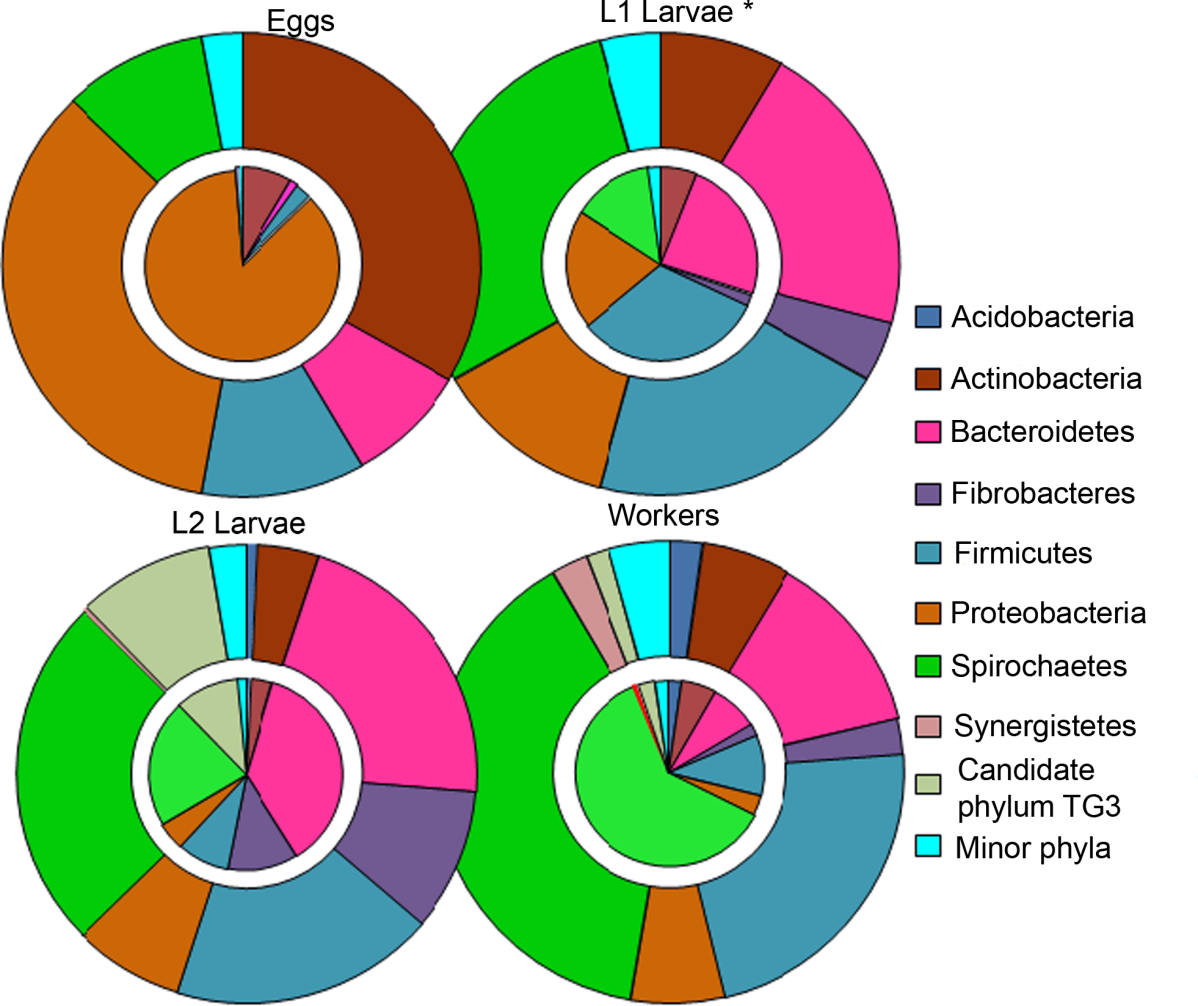
Phylum-level distribution of bacterial taxa from pyrotag libraries based on the 16S rRNA gene from eggs and the guts of the first instar (L1 larvae), second instar (L2 larvae) and workers of *N*. *arborum*. For each stage, the inner pie chart corresponds to the relative abundance of reads affiliated to each phylum. The outer pie chart corresponds to the number of OTUs for each phylum as a fraction of the total number of OTUs. *The charts for L1 larvae are based on 50 sequences excluding *Wolbachia* unlike the charts for eggs, L2 larvae and workers stages which are based on subsamples of 1367 reads. “Minor phyla” correspond to the pool of all phyla with <1% of reads and ≤ 5 OTUs in well sequenced libraries (eggs, L2 larvae, workers).

**Fig 2:**
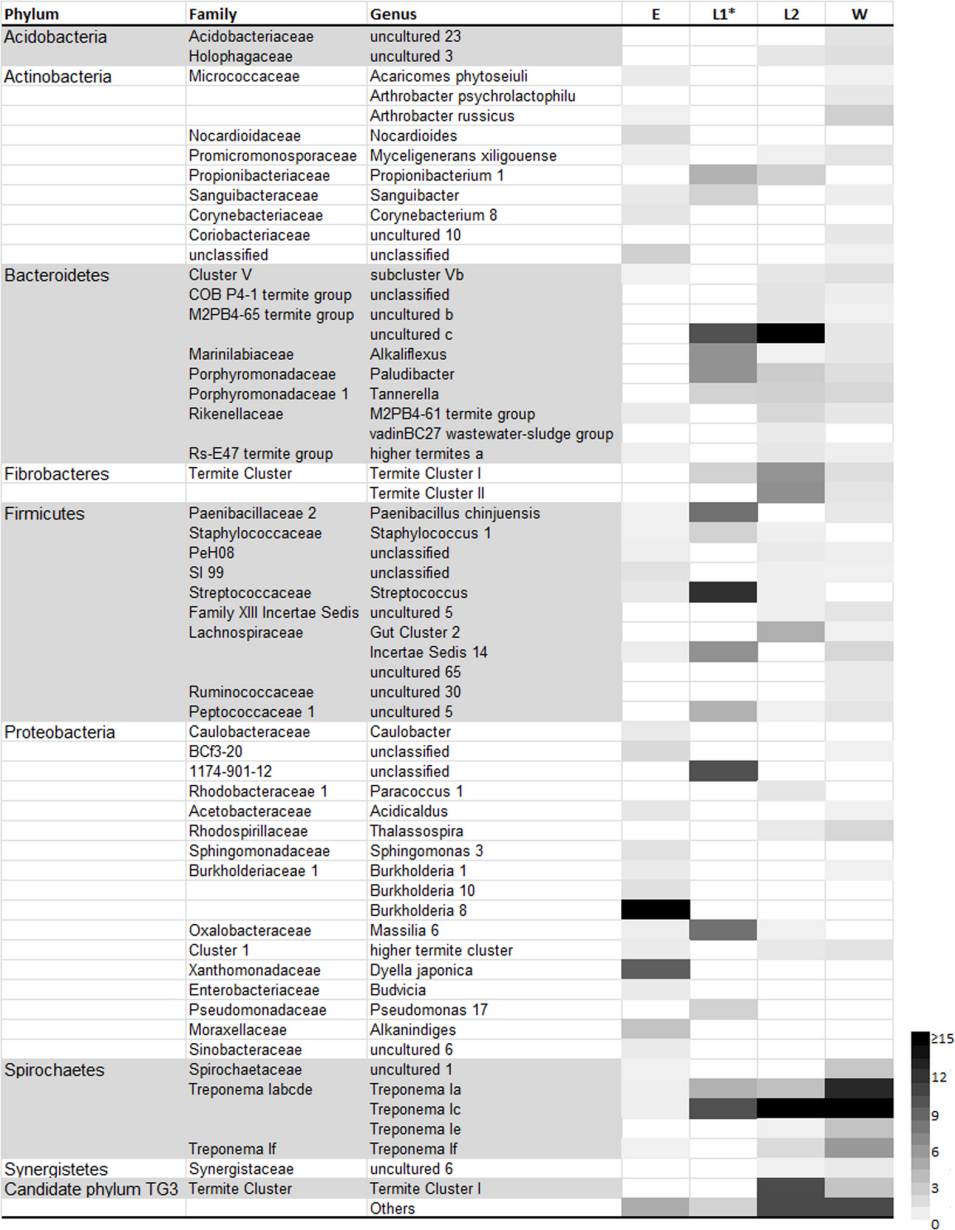
Relative abundances of the genus-level bacterial groups in eggs (E), the first instar larvae (L1), the second instar larvae (L2) and the worker caste (W) of *N*. *arborum*. Only genera > 0.5% in at least one stage are presented. The remaining genera are included in “Others”. The detailed classification with all the taxonomic levels is provided in the interactive spreadsheet (S1 Table). * The relative abundances for the L1 larval stage are based on 50 sequences excluding *Wolbachia*.

The bacterial community composition depended on the life stages of the termites. The eggs were dominantly colonized by Proteobacteria (86.25% of reads) and Actinobacteria (8.41% of reads) (Fig 1). Moreover, these two phyla accounted for nearly 70% of OTUs retrieved from this stage. Within Proteobacteria, the most abundant OTUs fell into the Burkholderiaceae (Betaproteobacteria) and Xanthomonadaceae (Gammaproteobacteria) families (Fig 2 and S1 Table). The remaining OTUs were affiliated to less abundant phyla, none of which accounted for more than 1.5% of reads with the exception of Firmicutes. The gut of L1 larvae were dominantly colonized by OTUs clustered with *Wolbachia*. After excluding these OTUs in downstream analyses, the remaining sequences fell primarily within Firmicutes (32% of reads), Bacteroidetes (24%), Proteobacteria (20%) and Spirochaetes (14%). Of Firmicutes, the most abundant sequences were affiliated to the genera *Streptococcus* (Lactobacillales) and *Paenibacillus* (Bacillales) while Bacteroidetes were mainly represented by the M2PB4-65 termite group, *Alkaliflexus* (Marinilabiaceae) and *Paludibacter* (Porphyromonadaceae). In the last larval stage, Bacteroidetes were more abundant (36.58% of reads and more than 20% of OTUs) and were from the M2PB4-65 Termite group accounting for 26.12% of reads. Besides Bacteroidetes, Spirochaetes (21.51%) mainly from *Treponema* I cluster, Fibrobacteres (12.00%) especially from the Termite subclusters I and II and the Candidate phylum TG3 (10.75%) were more abundant than in previous stages. In the worker caste, the majority of reads fell within the same phyla as for L2 larvae, but with a marked relative increase in the abundance of Spirochaetes (61.45% of reads and 39% of OTUs).

### OTUs common to the various life stages

**Fig 3:**
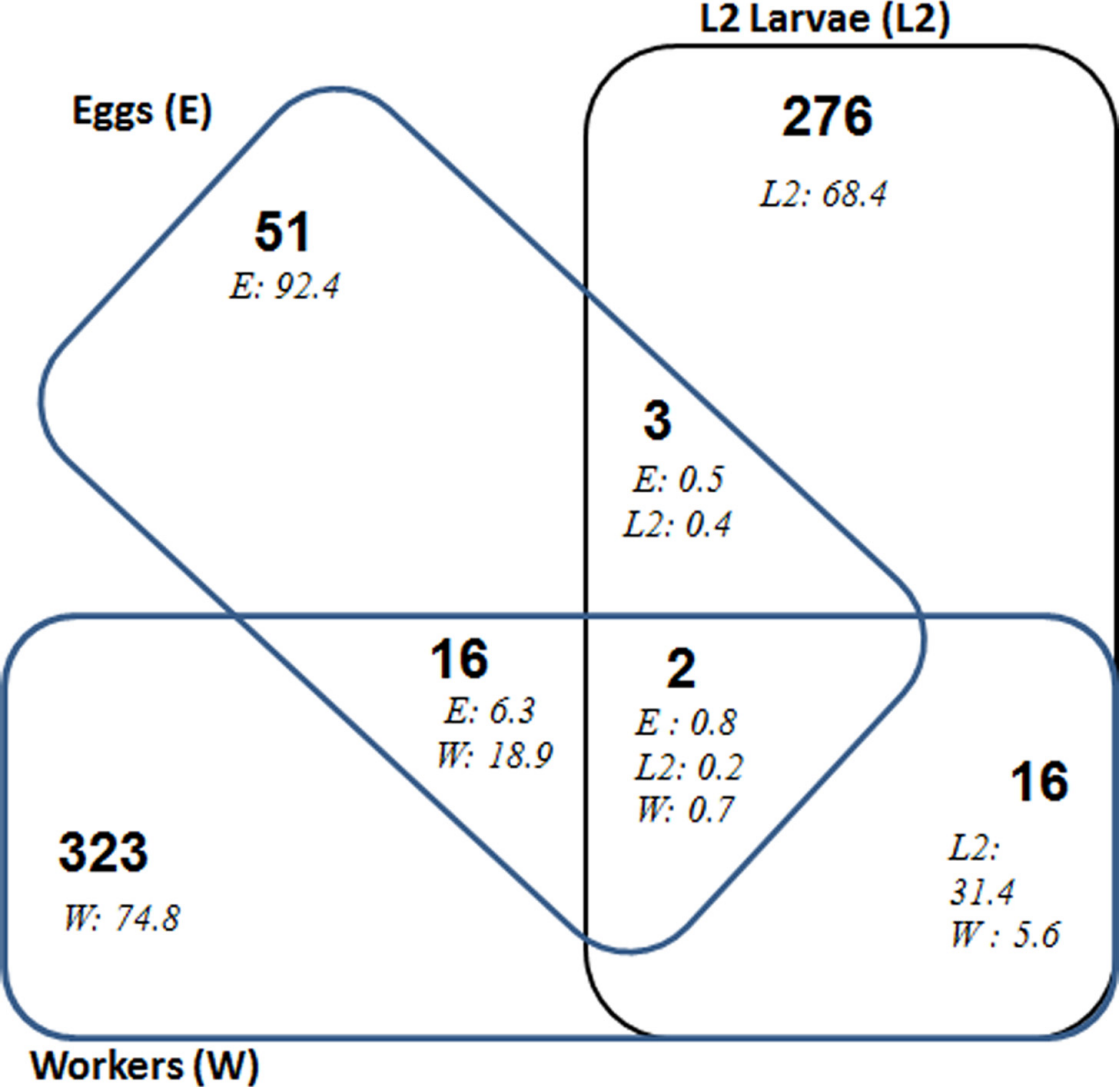
Venn diagram of bacterial OTUs (at 97% identity) common to the pyrosequencing libraries for eggs (E), L2 larvae (L2) and workers (W) of *N*. *arborum*. Numbers in bold indicate the number of OTUs and numbers in italics the relative abundance of the corresponding OTUs in each stage where they are detected. Details of the common OTUs are given in S2 Table.

Only two OTUs, accounting for less than 1% in all the pyrotag libraries, were common to the eggs, L2 larvae and worker caste termites of *Nasutitermes arborum* (Fig 3). These were Bacteroidetes affiliated to the M2PB4-61 Termite group (Rikenellaceae) and the higher termite a cluster (Rs-E47 termite group) respectively (S2 Table). Three OTUs were found in the eggs and L2 larvae and not in workers. They were from the genera *Staphylococcus* (Staphylococcaceae, Firmicutes), *Massilia*–6 (Oxalobacteraceae, Betaproteobacteria) and Subcluster Vb (Cluster Vb, Bacteroidetes) and together accounted for about 0.5% of reads in each of these stages. Sixteen OTUs were found in the eggs and worker caste termites but were not found in L2 Larvae. These common OTUs accounted for 6.3% and 18.9% of reads in eggs and workers respectively. They were primarily Firmicutes (5 OTUs, 1.76% or reads from eggs), Actinobacteria (4 OTUs, 2.71%) and Proteobacteria (4 OTUs, 1.39%). 16 OTUs were found in both L2 larvae and workers but not in other stages. There was a higher proportion of these common OTUs in L2 larvae (31.4% of reads), mainly from Bacteroidetes (10 OTUs, 24.36% of reads) especially from the M2PB4-65 termite group, from *Treponema* Ic cluster of Spirochetes (2 OTUs, 5.78% of reads in L2 larvae) and from Firmicutes (3 OTUs, 0.58% of reads in L2 larvae). These results indicate that there are significantly fewer OTUs in common between developmental stages than OTUs found only in one stage, which represented up to 92.4% of reads (or 70.53% of OTUs) from eggs, 68.4% reads (or 92.92% of OTUs) from L2 larvae, and 74.8% of reads (90.47% of OTUs) from the worker caste.

### Phylogenetic relationship between allopatric *Nasutitermes* species

**Fig 4:**
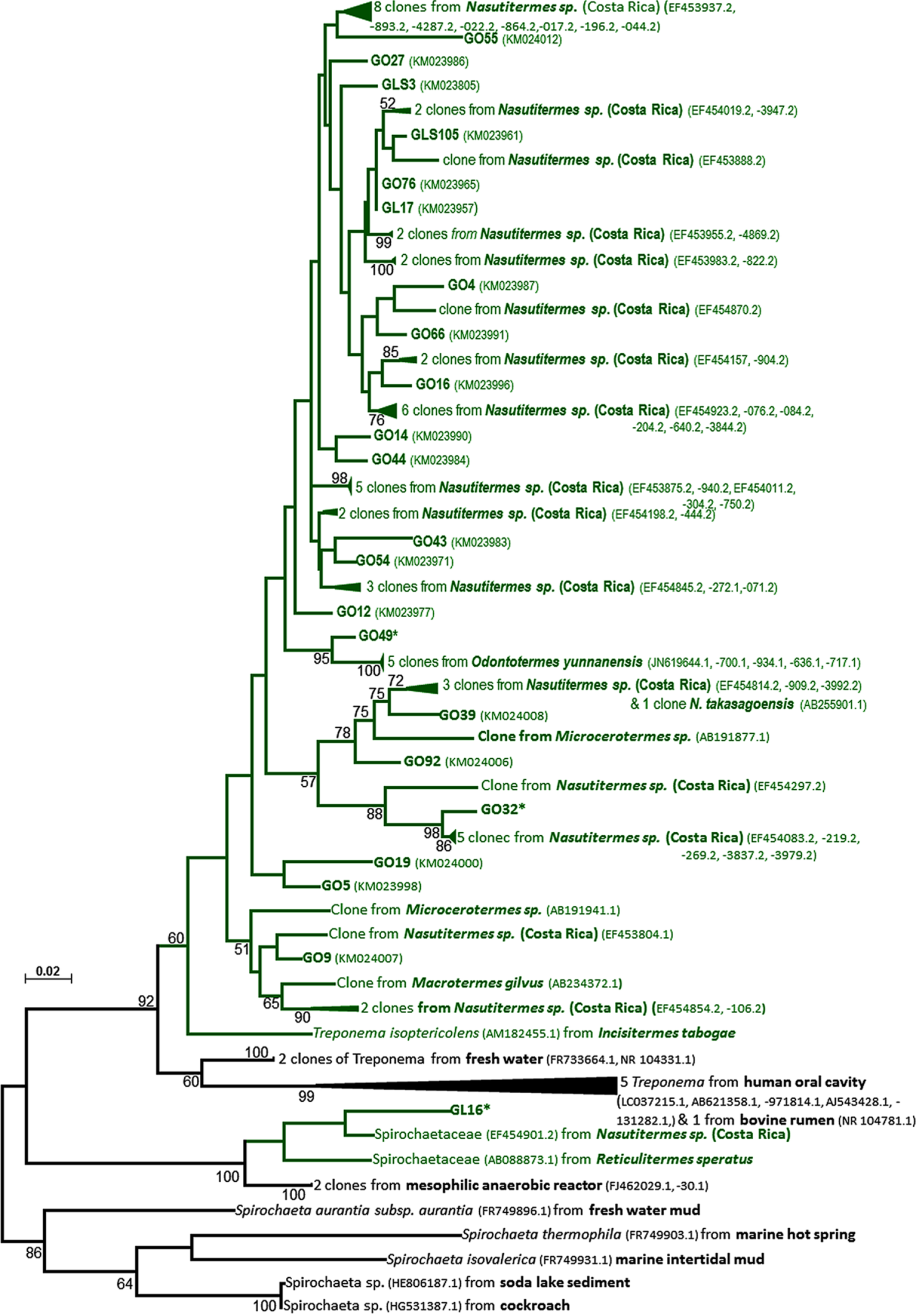
Phylogenetic tree illustrating the position of Spirochaetes-related OTUs from 16S rRNA gene libraries. The tree was constructed using the Maximum Likelihood-method implemented in MEGA version 6.0 and the Tamura-Nei model (analysis, 1000 bootstraps). Only bootstrap values >50% are presented. Clones from this study are referred to as GL and GLS (from the second instar larvae) and GO (from workers) and numbers in brackets correspond to NCBI accession numbers of sequences. * indicates sequences undergoing NCBI submission. Branches in green are entirely made of sequences from termites.

Owing to the longer reads, the 16S rRNA gene-based clone library allowed us to carry out a comparative analysis of the bacterial communities at a higher level of phylogenetic resolution. This approach generated 280 sequences from the four samples (eggs, L1, L2 and adult workers) including 253 high quality reads. To gain an insight into the role of the symbiont transmission route in the codiversification of the gut microbiota with the termite host, a phylogenetic analysis was carried out on the Spirochates-related OTUs, the dominant lineage from adult workers of *N*. *arborum* in comparison with previously published sequences available in databases. Most of the Spirochaetes-related OTUs clustered with those previously retrieved from the gut of allopatric *Nasutitermes* species (Fig 4).

## Discussion

There is still little information on the colonization of the termite gut by microorganisms since most previous studies of the gut microbiota have been limited to the worker caste. This is the first molecular survey of the microbial composition in eggs and larval stages of termites and provides information on whether the gut microbiota of later instars originates from transovarian transmission through the eggs or from early inoculation of the offspring.

The detection of bacteria from the eggs raised the question whether these bacteria were located within the egg. The eggs were surface-sterilized in 70% ethanol to check the hypothesis that termite symbionts are transmitted transovarially to the eggs. Unfortunately, the lack of an untreated control made it impossible to draw any conclusions on this point. Although some bacteria located on the surface of the eggs were certainly lysed by the 70% ethanol and washed away by sterile water, the presence of persistent contamination by bacteria or DNA should not be ruled out [19]. The presence of bacteria inside the egg was not unexpected as termite eggs have tiny channels through the outer lining of the eggs called micropyles [20]. The effectiveness of sterilizing the surface of the eggs using 70% ethanol in this study cannot be assessed and it is not possible, therefore, to conclude whether the bacteria detected were located on the surface or inside the eggs. Total DNA recovery from the eggs was of the same order of magnitude as in workers but 13–15 fold higher than in L1 and L2 larval stages. Although the concentration of DNA was adjusted to be equal for all samples prior to pyrosequencing analyses, this quantification only provided information on the pool of DNA extracted from egg tissue, *Wolbachia* and other microorganisms found in the sample. This study did not specifically quantify microbial DNA or the bacterial colonization density. Low bacterial density can have a significant effect on microbial profiling by pyrosequencing analyses [21,22]. Therefore, changes in relative abundance, especially in eggs and first instar L1, should be considered with caution since the dominance of *Wolbachia* at these host developmental stages means that the contribution of gut bacteria to the total DNA in the extracts was very small.

It was expected that termite-specific bacteria would colonize the embryos or the gut of larvae at an early stage, thus preventing the establishment of environmental lineages that would weaken the partner fidelity, as is the case for some insects [23]. Apart from *Wolbachia* (77.7% of reads in eggs), ubiquitous aerobic bacteria mainly affiliated to Proteobacteria and Actinobacteria were predominant in the eggs of *N*. *arborum*. The dominance of *Wolbachia* in the eggs as well in the first larvae and last instar is consistent with a previous study stressing the abundance of these maternally transmitted endosymbionts in tissues of immature stages such as larvae and pre-soldiers [24]. It is not clear whether the other bacteria found in the eggs were also vertically transmitted. To give a better estimate of termite-associated symbionts, a distinction was drawn between OTUs matching exclusively termite-specific phylotypes and OTUs matching free-living strains (S1 Fig). The dominant phyla in the eggs, Proteobacteria and Actinobacteria, consisted mainly of free-living lineages. Only two OTUs affiliated to the cluster–1 (Rs-K70 termite group, Deltaproteobacteria) and accounting for less than 0.5% in this stage were gut-specific symbionts, one of which was also found in all developmental stages. Fourteen other gut-specific OTUs amounting to not more than 2.5% mainly from Spirochaetes (*Treponema* and Uncultured 1 Spirochaetaceae) and Bacteroidetes (Subcluster Vb, Rs-E47 termite group, M2PB4-61 termite group) were found in the eggs. Overall, these findings suggest that eggs are predominantly colonized by bacteria acquired from the environment. The presence of a few gut-specific OTUs in the eggs may simply result from occasional transfers by workers, either during the frequent displacements *via* their salivarium, or during egg grooming by licking. Transfer during egg-laying by the queen might also be a source of bacterial contamination. The importance of these behavioral traits in termites was shown by the increase in egg size as a result of saliva transfer, and the failure of unlicked eggs to hatch [25]. It is possible that these interactions deposit gut-derived symbionts on the surface of eggs, followed by the ingestion of these bacteria by larvae. In this case, surface sterilization may have underestimated the gut derived bacteria in the eggs. The surface of the eggs was sterilized on the assumption that it was not a suitable environment for the survival of most termite-gut symbionts, most of which are anaerobic bacteria.

The high proportion of Proteobacteria and Actinobacteria could have a functional significance as their defensive role in eggs or larvae has been reported in soil and marine invertebrates [26–30]. The largely dominant phylotype in the eggs was affiliated to *Burkholderia* (Betaproteobacteria) some of whose members have been reported to protect leaf-cutting ants against entomopathogenic fungi [31]. The functions of Proteobacteria and Actinobacteria in termite eggs have not yet been documented.

In the first instar larvae, there was a relative increase of Firmicutes, Bacteroidetes, Spirochaetes and the emergence of Fibrobacteres in addition to Actinobacteria and Proteobacteria detected in the eggs, indicating the diversification of the community at this developmental stage. Of a particular interest was the relative increase in specific termite-gut OTUs, covering up to 32% of reads, most of which were closely related to *Treponema* subclusters Ic and Ia (Spirochaetes), *Alkaliflexus*, *Paludibacter* and *Tannerella* (Bacteroidetes). In the last instar larvae, additional phyla were detected, including the candidate phylum TG3 (mostly TG3 subphylum 1). Although TG3 members are found widely in different environments, those from the termite gut are autochthonous lineages constituting a monophyletic cluster [32]. The overall gut-specific bacteria (89.6% of reads) were largely dominant at that stage, confirming the clear trend observed in the previous stage. In worker caste termites, the bacterial community was dominated by Spirochaetes, Firmicutes, Bacteroidetes already present in the last instar larvae. The termite-gut specific bacteria found at this stage accounted for a similar percentage of reads as in the last instar larvae (86.6% of reads). There was a high overlap of genus-level taxa specific to the termite gut between worker caste and the last instar larvae.

Overall, these findings indicated that the last instar larvae, although still dependent on workers for feeding, had already been colonized by the bulk of the gut bacterial symbionts. This suggests that the acquisition and perpetuation of core gut microbiota through host generations rely on social exchanges, allowing the transmission of strict anaerobic gut bacteria such as Fibrobacteres and Spirochaetes. Since the pioneer work of Grassé, stomodeal exchange is considered as the major route for transferring food to offspring of higher termites [33,34]. Unlike lower termites, proctodeal trophallaxis has not as yet been demonstrated in higher termites. Strict anaerobic symbionts of termites are unable to survive outside the host and have to be transmitted by direct ingestion of excreta from adult individuals either by trophallaxis or by coprophagy. Recently, Köhler and colleagues [35] have shown similarities in the bacterial community of the rectum and the anterior gut in wood-feeding higher termites belonging to *Nasutitermes* genus. Of particular interest are the presence and the high relative abundance of Fibrobacteres and Spirochaetes in the crop and the rectum of *Nasutitermes corniger*. Based on these observations, Köhler and colleagues [35] suggested that the fecal material is consumed by the termites. Our results supported the hypothesis that, in *Nasutitermes* species, strict anaerobic bacteria (Fibrobacteres and Spirochaetes) are more probably transferred via the fecal route or via food regurgitated from the crop. This hypothesis is relevant since the earlier development stages (egg and instar larvae L1) were dominantly colonized by *Wolbachia* which is an intracellular termite symbiont, the contribution of gut bacteria (including Fibrobacteres and Spirochetes) being very small. Another point of particular interest in the present study was the marked difference in the relative abundances of each phylum between instar larvae L2 and workers. In last instar larvae, termite gut-specific lineages were mostly from Bacteroidetes, Fibrobacteres and TG3 as well as Spirochaetes, whereas Spirochaetes dominated in the worker caste termites. The dominance of Spirochaetes in the worker caste is in agreement with previous studies that showed this phylum to be the main bacterial group, accounting for more than the half of the total bacterial community in wood-feeding higher termites (reviewed by [7]). The variation in the relative abundance through the development stages with the dominance of anaerobes in the final stages might be explained by the physical and chemical conditions in the gut. The gut of worker caste termites has structured microenvironments with fundamentally different physical and chemical conditions as well as different microbial processes [7]. In a comprehensive study linking physical and chemical conditions with the structure of the microbial communities in the different gut compartments of the wood-feeding higher termite, *Nasutitermes* spp., it was reported that the dilated hindgut paunch was the only anoxic gut region and harbored a dense community of Spirochaetes and Fibrobacteres while other gut compartments had small but distinct populations specific to each gut region [35]. The higher relative abundance of anaerobes in the final developmental stages may, therefore, stem from environmental conditions that are more favorable for the growth of the corresponding groups, but further investigation of the physical and chemical conditions in the gut of larvae is required to confirm this hypothesis.

The change in relative abundance of gut-specific lineages may be related to the change in diet across life stages as diet appears to influence the relative abundance, but not membership of the gut communities [9]. In higher termites, the main social exchange of food with the brood is stomodeal trophallaxis where workers provide nitrogen-rich regurgitates made of pure saliva for the youngest instars, then progressively loaded with wood particles from the gizzard for older larval stages [34,36]. Our results consistently suggest that the succession of bacterial communities tends towards the promotion of symbionts capable of degrading wood components through the developmental stages of *N*. *arburum*. Many bacterial genes encoding glycoside hydrolases putatively involved in the degradation of cellulose and hemicellulose have been detected in the hindgut of *Nasutitermes* spp.[37,38]. Diverse endoglucanases and nitrogenases were putatively assigned to both *Treponema* (Spirochaetes species) and Fibrobacteres while diverse endoxylanases and glycosidases were only assigned to *Treponema* [37].

A phylogenetic comparison of Spirochaetes-related OTUs, the major lineage in adult workers of *N*. *arborum*, with previous published sequences available in databases indicated that the sequences retrieved in the present study mainly clustered with those from allopatric *Nasutitermes* species. This indicates that the gut symbionts are not randomly acquired from the environment, but are termite gut-specific [4,9]. The composition of the gut microbiota in the worker caste of *N*. *arborum* was in line with that of congeneric species previously reported (S3 Table) [6,32,35,39]. These observations suggest that the transmission of symbionts to offspring through social exchanges plays a key role in the codiversification of the gut microbiota with the termite host.

## Conclusion

This first study of the microbiota of flagellate-free termites throughout their life stages demonstrated that, despite the strong relationship with their host, the bacterial colonization of offspring is a gradual process becoming very conspicuous at the last larval stage. The large proportion of lineages specific to termites in the gut of later larval stages, before the self-feeding worker stage, is clear evidence of the acquisition of the corresponding taxa through social exchanges. Further research is required to provide more precise information about the mechanisms of bacterial colonization. The compositional gaps still existing between larval stages on the one hand and between larvae and the climax state achieved in workers on the other hand emphasize the significance of feeding behavior in the establishment of microbial lineages and demonstrates the complexity of interactions between all members of a termite colony in general, and between feeders (workers) and the brood in particular. The similarities between the gut microbiota in worker caste termites of *N*. *arborum* and other species in the same genus indicate that this is a long-lasting mutualistic association.

## Supporting Information

S1 Fig Ternary plot of the distribution termite-gut specific genus-level taxa between eggs, the second instar larvae and worker caste of *N*. *arborum*. The size of symbols is proportional to the number of reads associated with the taxon. Only genera with ≥ 0.5% of reads in at least one stage are distinguished. Those below 0.5% are pooled in “Others.
(TIF)

S1 Table Relative abundance and number of OTUs of bacterial taxa in pyrotag libraries from eggs (E), the gut of the first instar larvae (L1), the second instar larvae (L2) and workers (W) of *N*. *arborum*. *The relative abundances are calculated for L1 larvae on the basis of 50 reads as compared to 1367 reads for other stages. Clicking on the signs (+) or (-) expands or collapses the taxonomic ranks of the lineages (phylum, class, order, family and genus).
(XLSX)

S2 Table Classification and abundances of OTUs shared between eggs (E), the second instar larvae (L2) and worker caste (W) of *N*. *arborum*. + indicate OTUs whose best hits are sequences from the termite gut habitat.
(DOCX)

S3 Table Comparison of the relative abundance of the five major bacterial phyla in the gut of workers of *N*. *arborum* (Na) with the microbiotas of *N*. *corniger* (Nc) and *N*. *takasagoensis* (Nt) from other studies either by pyrosequencing (Dietrich et al. 2014; Köhler et al. 2012) or cloning (Hongoh et al. 2006; Miyata et al. 2007). nd indicates values of relative abundance which were not determined
(DOCX)
