# Supplementary figures and images for: Profiling the Succession of Bacterial Communities throughout the Life Stages of a Higher Termite Nasutitermes arborum (Termitidae, Nasutitermitinae) Using 16S rRNA Gene Pyrosequencing
Source: PLoS One. 2015 Oct 7;10(10):e0140014. doi: 10.1371/journal.pone.0140014 (PMC4596844; doi:10.1371/journal.pone.0140014)

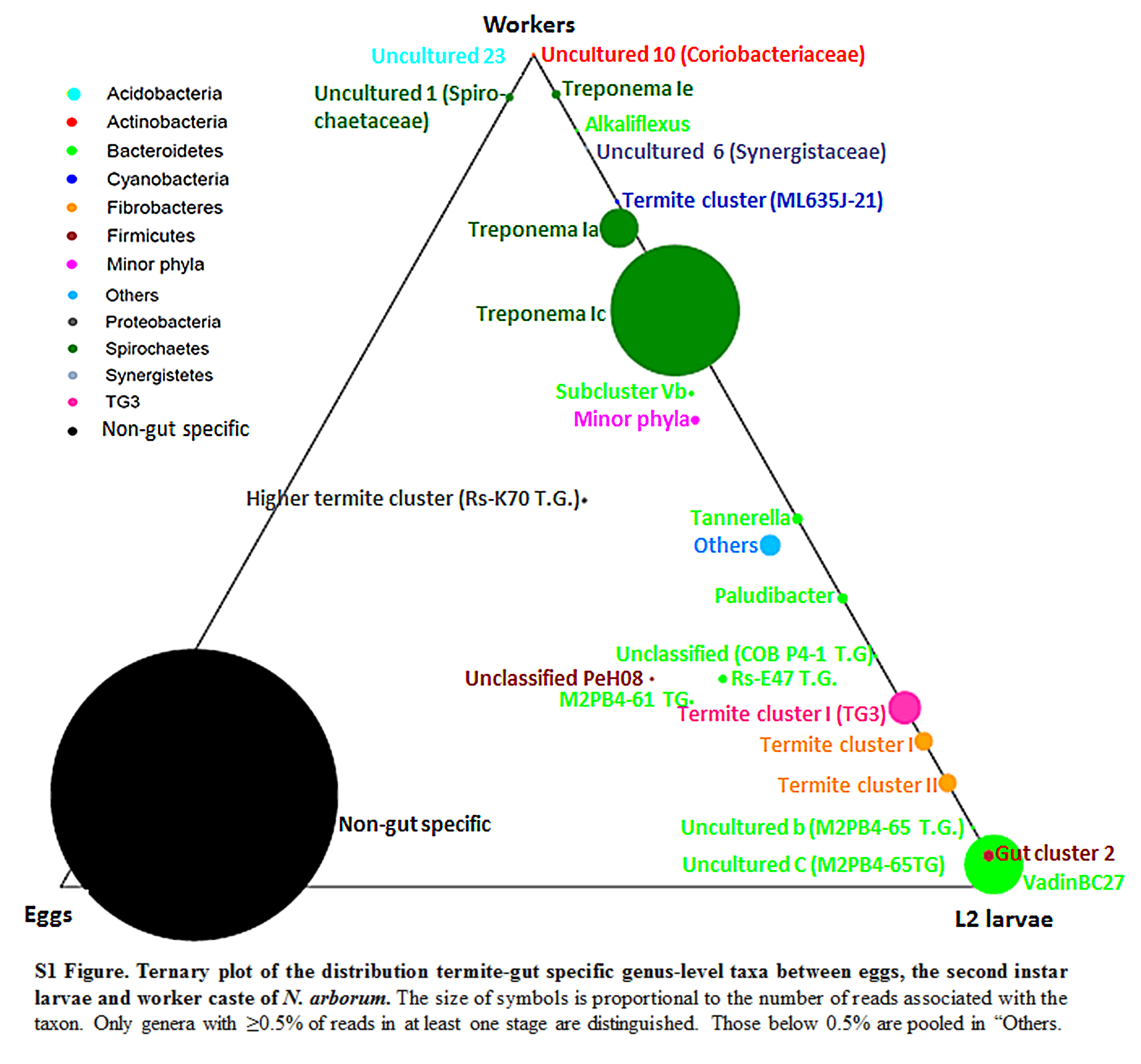

Supplement: S1 Fig — The size of symbols is proportional to the number of reads associated with the taxon. Only genera with ≥ 0.5% of reads in at least one stage are distinguished. Those below 0.5% are pooled in “Others. (TIF) [file pone.0140014.s001.tif]
