# Supplementary material for: Profiling the Succession of Bacterial Communities throughout the Life Stages of a Higher Termite Nasutitermes arborum (Termitidae, Nasutitermitinae) Using 16S rRNA Gene Pyrosequencing
Source: PLoS One. 2015 Oct 7;10(10):e0140014. doi: 10.1371/journal.pone.0140014 (PMC4596844; doi:10.1371/journal.pone.0140014)
Supplement: S3 Table — nd indicates values of relative abundance which were not determined (DOCX) [file pone.0140014.s004.docx]

**S3 Table. Comparison of the relative abundance of the five major bacterial phyla in the gut of workers of *N. arborum* (Na) with the microbiotas of *N. corniger* (Nc) and *N. takasagoensis* (Nt) from other studies either by pyrosequencing (Dietrich et al. 2014; Köhler et al. 2012) or cloning (Hongoh et al. 2006; Miyata et al. 2007)**.

|  | Pyrosequencing | | | | | Cloning | |
| --- | --- | --- | --- | --- | --- | --- | --- |
| Phylum | This study | Dietrich et al. 2014 | | Kohler et al. 2012 | | Hongoh et al. 2006 | Miyata et al. 2007 |
|  | Na | Nc | Nt | Nc | Nt | Nt | Nt |
| Actinobacteria | 5.85 | 0.81 | 1.5 | 0.89 | 1.54 | nd | 0.00 |
| Bacteroidetes | 8.34 | 6.36 | 5.28 | 4.72 | 5.42 | 4.70 | 8.33 |
| Fibrobacteres | 2.12 | 9.97 | 0.04 | 8.15 | 0.04 | 14.10 | 5.83 |
| Firmicutes | 10.46 | 6.69 | 12.85 | 6.70 | 12.48 | nd | 9.79 |
| Proteobacteria | 3.51 | 4.04 | 2.24 | 2.97 | 2.38 | nd | 3.13 |
| Spirochaetes | 61.45 | 55.51 | 69.33 | 67.550 | 69.0 | 57.10 | 62.10 |
| Synergistetes | 0.80 | 0.06 | 0.10 | 0.73 | 0.09 | nd | nd |
| Candidate division TG3 | 2.85 | 13.05 | 6.17 | 4.05 | 6.5 | 9.4 | 4.58 |

nd indicates values of relative abundance which were not determined
