# Supplementary material for: Profiling the Succession of Bacterial Communities throughout the Life Stages of a Higher Termite Nasutitermes arborum (Termitidae, Nasutitermitinae) Using 16S rRNA Gene Pyrosequencing
Source: PLoS One. 2015 Oct 7;10(10):e0140014. doi: 10.1371/journal.pone.0140014 (PMC4596844; doi:10.1371/journal.pone.0140014)
Supplement: Supplementary file 4 [file pone.0140014.s003.docx]

**S2 Table. Classification and abundances of OTUs shared between eggs (E), the second instar larvae (L2) and worker caste (W) of *N. arborum*.** + indicate OTUs whose best hits are sequences from the termite gut habitat

| Phylum | Family | Genus | E | L2 | W | | OTUs | % identity | Ac. Number | Isolation source |
|---|---|---|---|---|---|---|---|---|---|---|
| Acidobacteria | Holophagaceae | uncultured-3 | 0 | 0.66 | 1.24 | + | 16 | 100% | AB255927.1 | Nasutitermes takasagoensis |
| Actinobacteria | Sanguibacteraceae | Sanguibacter | 0.66 | 0 | 0.37 | | 36 | 96% | JF911067.1 | bulk soil |
| | Micrococcaceae | Arthrobacter-russicus | 0.15 | 0 | 0.44 | | 70 | 93% | JQ071627.1 | semiarid desert soil |
| | Promicromonosporaceae | Myceligenerans-xiligouense | 0.22 | 0 | 0.37 | | 72 | 90% | JX638862.1 | mastitic milk |
| | unclassified | unclassified | 1.68 | 0 | 0.15 | | 20 | 95% | HM295099.1 | skin, volar forearm |
| Bacteroidetes | Cluster-V | subcluster-Vb | 0 | 0.44 | 0.29 | + | 54 | 97% | AB255909.1 | Nasutitermes takasagoensis |
| | | | 0 | 0.07 | 0.22 | + | 167 | 100% | AB255909.1 | Nasutitermes takasagoensis |
| | | | 0 | 0.07 | 0.07 | + | 250 | 97% | AB255909.1 | Nasutitermes takasagoensis |
| | | | 0.07 | 0.07 | 0 | + | 237 | 98% | AB191994.1 | Microcerotermes sp. 1 |
| | M2PB4-65-termite-group | uncultured-c | 0 | 21.43 | 0.22 | + | 2 | 96% | AY571426.1 | Reticulitermes santonensis |
| | Porphyromonadaceae | Paludibacter | 0 | 0.15 | 0.8 | + | 38 | 99% | AB243251.1 | Microcerotermes sp. 2 |
| | | Tannerella | 0 | 1.39 | 0.66 | + | 14 | 100% | AB255914.1 | Nasutitermes takasagoensis |
| | | Tannerella | 0 | 0.07 | 0.22 | + | 138 | 93% | AB255914.1 | Nasutitermes takasagoensis |
| | Rikenellaceae | M2PB4-61-termite-group | 0 | 0.07 | 0.15 | + | 193 | 97% | AB255910.1 | Nasutitermes takasagoensis |
| | | | 0.51 | 0.15 | 0.51 | + | 29 | 92% | AB192001.1 | Microcerotermes sp. 1 |
| | Rs-E47-termite-group | higher-termites-a | 0 | 0.37 | 0.07 | + | 85 | 99% | EF454900.2 | Nasutitermes sp. (Costa R.) |
| | | | 0 | 0.29 | 0.07 | + | 121 | 99% | AB255906.1 | Nasutitermes takasagoensis |
| | | | 0.29 | 0.07 | 0.15 | + | 80 | 96% | EF454472.2 | Nasutitermes sp. (Costa R.) |
| Candidate division SR1 | unclassified | unclassified | 0.15 | 0 | 0.15 | + | 157 | 98% | JF917298.1 | Zootermopsis sp. |
| Chloroflexi | Anaerolineaceae | uncultured-11 | 0.22 | 0 | 0.44 | | 61 | 93% | KC432692.1 | cecal content from mice |
| Firmicutes | Staphylococcaceae | Staphylococcus-1 | 0.15 | 0.22 | 0 | | 113 | 100% | KF094310.1 | skin, antecubital fossa |
| | PeH08 | unclassified | 0 | 0.29 | 0.07 | + | 123 | 97% | EF454727.2 | Nasutitermes sp. (Costa R.) |
| | | | 0.15 | 0 | 0.15 | + | 161 | 95% | EF454749.2 | Nasutitermes sp. (Costa R.) |
| | Sl-99 | unclassified | 1.02 | 0 | 0.07 | | 33 | 89% | AB571484.1 | vegetable |
| | Lachnospiraceae | Gut-Cluster-2 | 0 | 0.22 | 0.07 | + | 164 | 89% | AB243283.1 | Microcerotermes sp. 2 |
| | | Incertae-Sedis-14 | 0.37 | 0 | 0.95 | | 27 | 93% | KF507370.1 | Mouse skin |
| | Peptococcaceae-1 | uncultured-5 | 0 | 0.07 | 0.29 | + | 129 | 92% | JN620049.1 | Odontotermes yunnanensis |
| | Peptococcaceae-3 | Sporotomaculum-hydroxybenz | 0.15 | 0 | 0.07 | + | 184 | 89% | EF454544.2 | Nasutitermes sp. Costa R. |
| | Erysipelotrichaceae | Turicibacter | 0.07 | 0 | 0.66 | | 55 | 92% | KC432692.1 | cecal content from mice" |
| Proteobacteria | Acetobacteraceae | Acidicaldus | 0.8 | 0 | 0.07 | | 42 | 99% | JQ383580.2 | FACE soil sample |
| | Oxalobacteraceae | Massilia-6 | 0.29 | 0.15 | 0 | | 89 | 100% | KF101847.1 | skin, volar forearm |
| | Cluster-1 | higher-termite-cluster | 0.37 | 0 | 0.07 | + | 99 | 100% | EF454405.2 | Nasutitermes sp.(Costa R.) |
| | | | 0.07 | 0 | 0.29 | + | 128 | 96% | AB255924.1 | Nasutitermes takasagoensis |
| Spirochaetes | Spirochaetaceae | uncultured-1 | 0.15 | 0 | 1.76 | + | 17 | 94% | AB255904.1 | Nasutitermes sp.(Costa R.) |
| | Treponema-Iabcde | Treponema-Ic | 0 | 5.56 | 0.07 | + | 7 | 96% | AB255967.1 | Nasutitermes takasagoensis |
| | | | 0 | 0.22 | 1.1 | + | 26 | 95% | AB255888.1 | Nasutitermes takasagoensis |
| | | | 0.07 | 0 | 12.95 | + | 3 | 96% | EF454958.2 | Nasutitermes sp.(Costa R.) |
| Total | | | 7.61 | 32.04 | 25.24 | | | | | |
